# Epidemiology of Alcohol Use in Late Adolescence in Greece and Comorbidity with Depression and Other Common Mental Disorders

**DOI:** 10.1155/2019/5871857

**Published:** 2019-09-25

**Authors:** Grigorios Michalis, Stefanos Bellos, Spyridon Politis, Konstantina Magklara, Petros Petrikis, Petros Skapinakis

**Affiliations:** Department of Psychiatry, University of Ioannina School of Medicine, Ioannina, Greece

## Abstract

**Background:**

The aim of the current study was to examine the prevalence and associations of hazardous alcohol use with sociodemographic variables and its comorbidity with depression and other common mental disorders in a sample of Greek adolescents between 16 and 18 year old.

**Methods:**

We recruited 2431 adolescents attending 25 senior high schools in Greece. We assessed depressive and anxiety disorders using the computerized version of a fully-structured psychiatric interview (the revised Clinical Interview Schedule / CIS-R). Alcohol use was assessed using questions taken from a previous WHO school survey.

**Results:**

Approximately one-third of adolescents (overall: 30.7%, boys: 39.2%, girls: 21.9%, p < 0.001) consumed alcohol on a weekly basis. The experience of excessive consumption, leading to drunkenness at least two or more times in their lifetime, was reported by 15.39% of the adolescents (19.42% for the boys and 11.24% for the girls, p < 0.001). Frequent alcohol consumption and drunkenness were strongly associated with the presence of depression, all other anxiety disorders except panic disorder, current smoking, and lifetime cannabis use, lower school performance, bad or fair relationship with parents, and increased health services use.

**Conclusion:**

Alcohol use is highly prevalent among Greek adolescents. Special attention for the development of more focused preventive strategies should be paid to adolescents suffering from depression or other common mental disorders.

## 1. Introduction

The onset of alcohol use and alcohol-related problems mainly occur during adolescence [1, 2]. The way alcohol is consumed at this period of life, including age of onset, quantity, and frequency of use, is strongly associated with alcohol related behaviors later in adulthood [3]. Alcohol is a major risk factor for morbidity and mortality in youth, as 1 out of 4 deaths among aged 15–29 in the developed world is associated with alcohol use [4]. Furthermore, alcohol-related problems have been associated with physical and mental health problems, psychosomatic symptoms, risky and violent behaviors, low achievement in school, road accidents, and injuries [5–7].

Data from large international epidemiological studies, although very useful for cross-cultural comparisons do not study extensively hazardous alcohol consumption and its associations with sociodemographic variables and comorbidity with other common mental disorders in this age period [8, 9]. The association between hazardous alcohol consumption and those determinants are useful for informing etiopathology of alcohol-related problems or planning prevention strategies. Furthermore, these associations may significantly differ across different cultures of alcohol consumption, especially between Southern and Northern Europe countries. Thus, reliance on data about those associations from countries with different alcohol-related culture may be inconclusive. Based on the above, the more detailed study at the country level of the prevalence of hazardous alcohol use and its association with depression or anxiety disorders would be of special importance for planning culture-specific prevention strategies and would lead to useful conclusions about the possible differences in risk-factors and consequences of alcohol-related problems across cultures.

The aim of the current study was to assess the prevalence of hazardous alcohol use with depression and anxiety disorders, a range of school and family-related variables, and the use of health services in a sample of Greek adolescents aged 16–18 and attending senior high schools. Previous studies originating from Greece had generally focused on estimating the prevalence of alcohol-behaviors without taking into account the association with depression or anxiety disorders [10, 11]. The results of our research will fill the gap in the current scientific literature by providing useful information for the planning of alcohol prevention programs for teenagers. In addition, our study was conducted shortly before the beginning in 2009 of the current socioeconomic crisis in Greece. We therefore considered it important to investigate some factors related to the drinking habits of alcohol by Greek teenagers during the critical period that preceded the beginning of the crisis presenting data that can be compared with corresponding data that will be reported after the crisis.

## 2. Methods

### 2.1. Description of the Data Set

We used data from a previous cross-sectional epidemiological survey carried out in secondary schools in Greece [12]. A convenient sample of 25 schools from Athens, the North-Western Part of Greece and one island in the Aegean sea (Paros) took part in the study. The aim of the original project was to investigate the prevalence and associations of common mental disorders in 16–18 year old adolescents. Further sample and methodological details of the survey are available elsewhere [12].

### 2.2. Design of the Study and Data Collection Procedure

We obtained written consent for the participation in the study from both parents and students. We used a two-phase sampling design to select students in our study [13]. In the first phase, all students (*N* = 5,614, response rate 82%) completed the screening questionnaire of the Revised Clinical Interview Schedule (CIS-R) (see next section). To include students for the second phase we used a stratified random sampling procedure according to the scores on the screening questionnaire: 100% of those scoring high on the screening instrument (above 75th percentile), 30% of those scoring in the middle and 10% of those scoring low (below 25th percentile). In the second phase (*N* = 2, 431, response rate 95%) students self-completed the computerized version of a fully-structured psychiatric interview (CIS-R, see next section). The main fieldwork took place between January 2007 and December 2008.

### 2.3. Assessment of Depression and Anxiety Disorders

In order to assess common mental disorders we used the revised Clinical Interview Schedule (CIS-R) [14, 15]. The CIS-R has been validated in the Greek adolescent population on the same dataset [12]. In our study we used the computerized version of the CIS-R which has been found to be comparable with the regular interview [16]. The participated pupils completed the interview in the computer laboratories of their schools. More details on the use of CIS-R in this sample and the assessment of psychiatric morbidity are available elsewhere [17, 18]. All psychiatric disorders were defined according to ICD-10 research diagnostic criteria using specifically designed diagnostic algorithms [17, 18].

### 2.4. Assessment of Alcohol Consumption

We assessed alcohol consumption, using the questions of a previous WHO study, asking about the frequency of drinking beer, wine, or spirits, separately [19]. The possible answers were “never,” “rarely,” “at least every month,” “at least every week,” or “daily”. We then grouped alcohol use into three categories (never, monthly or less, at least weekly). The adolescents were also asked a question about excessive consumption leading to drunkenness: “Have you ever had so much alcohol that you were really drunk?” and the possible answers were “Never,” “once” and “2 times or more.” “Drunkenness” was defined as having a history of having been drunk 2 or more times in a lifetime.

### 2.5. Socioeconomic, Sociodemographic and Other Variables

We collected information on gender, age, parental marital status, academic performance, mother's and father's educational status, mother's and father's employment status, family's financial condition, and relationship with mother and father. Since age roughly corresponds with grade (10^th^ grade: 16 years-old, 11^th^: 17 years-old, 12^th^: 18 years-old) we used grade instead of age in our analyses. We also assessed the use of health services during the past 12 months, defined as having visited a) a family doctor for any medical reason ≥3 times during the past 12  months or b) a family doctor or a mental health professional for a psychological reason at least once during the previous 12  months. Details on the methodology we have used to assess these variables are given elsewhere [17, 18].

### 2.6. Statistical Analyses

The analyses were all conducted using STATA 12.0. To take into account the complex design of our study we used the survey command in STATA (“svy” prefix). These STATA commands take into account the stratification, clustering, and weighting aspects of our sampling design in order to calculate more accurate point estimates and standard errors that are larger (i.e., more conservative) compared to the nonsurvey methods. We used the default variance estimation method which uses the first-order Taylor series linear approximation [20]. These variance estimators are analogous to the robust estimators also known as Huber/White estimators [20]. Due to the stratified random sampling we have also applied probability weights. For this reason, although all numbers reported are actual numbers, the percentages are weighted. It should be noted that the effect of schools was negligible with an intraclass correlation coefficient close to zero (<0.08). For this reason, it was not necessary to use a multilevel model (level 1: individuals, level 2: schools) using the corresponding STATA commands and we preferred the more widely used survey commands as noted before [20].

To investigate the association between frequent alcohol drinking/getting drunk and the various sociodemographic variables we used logistic regression modelling with the “logistic” command in STATA (using the “svy” prefix) . For each dependent variable we (alcohol drinking or getting drunk) we derived two models: model 1—the crude model with only the dependent and each sociodemographic variable as the covariate. Model 2 included all other sociodemographic variables simultaneously in order to adjust for confounding. For statistical significance we used the conventional level of *p* < 0.05, however, due to the multiple comparisons on Table 1 we also indicated the variables significant at a value of *p* < 0.01. For the comorbidity analyses the dependent variable was the comorbid condition (e.g., depression), while alcohol-related conditions was entered as an independent binary variable. In these analyses we only adjusted for age and sex to avoid over-adjustment. Use of health services was also investigated using models analogous to the comorbidity models.

## 3. Results

### 3.1. General Description of the Sample

5614 pupils were screened in the first phase and a stratified random sample of 2431 was selected for a detailed interview in the second phase (59% girls, 39% 10th grade, 32% 11th grade, 29% 12th grade).

### 3.2. Frequency of Alcohol Consumption

The frequency of alcohol consumption by gender and type of alcohol beverage is presented in Table 1. Overall 30.7% of students reported the use of any type of alcohol at least weekly (39.2% for male versus 21.9% for female, *p* < 0.001). Lifetime drunkenness (getting drunk 2 times or more) was reported by 15.4% of students and was also higher in males (19.4% versus 11.2%, respectively, *p* < 0.001). Regarding the correlation between increased consumption and drunkenness, 38.7% of those consuming alcohol at least weekly have got drunk 2 or more times in their lifetime, while 73.6% of those who had been drunk at least 2 or more times in their lifetime were also drinking at least weekly.

### 3.3. Association of Alcohol-Related Problems with Sociodemographic and Socioeconomic Variables

Table 2 illustrates the association between weekly alcohol consumption and lifetime drunkenness (getting drunk 2 times or more) and a range of sociodemographic/socioeconomic variables. In the fully-adjusted model we found statistically significant associations for both alcohol-related variables for male gender, increasing age (class grade), lower school performance, and not good relationship with the father. A not good relationship with mother was associated with weekly alcohol consumption, while students with a self-employed father were more likely to report drunkenness. The presence of financial difficulties showed evidence of an inverse U-shaped type of association with the medium level of difficulties having higher association with both problematic alcohol-behaviors. In the above analysis, the inclusion of the other variable as an independent co-variant (e.g., “drunkenness” in models where “frequency alcohol consumption” was used as the dependent variable and, correspondingly, “frequency alcohol consumption” in models where “drunkenness” was considered as the dependent variable) did not substantially modify the presented associations.

### 3.4. Comorbidity of Alcohol-Related Problems

Table 3 presents the results of our comorbidity analysis. Current depression was significantly associated with both alcohol-related variables with odds ratios greater than 2. Analogous findings were also observed for all anxiety disorders except panic disorder. Use of other substances was also higher compared to controls. Suicidal ideation was also increased.

### 3.5. Use of Health Services

Table 4 presents the result of the use of health services. Use of general medical services was significantly higher in both alcohol-related variables, but the association with mental health visits was statistically significant only for drunkenness.

## 4. Discussion

### 4.1. Main Findings

The majority of the adolescents in our sample drink alcohol monthly or less while a substantial proportion of adolescents use alcohol in a possibly hazardous way (on at least weekly basis or they have got drunk ≥2 times in their lifetime). Male sex, older age, bad relationship with parents (mainly with the father), low school performance, and smoking cigarettes or using cannabis are the variables which are stronger associated with hazardous alcohol use. Moreover, hazardous alcohol consumption was strongly associated with depression and other anxiety disorders, suicidality, and increased use of general health services.

Regarding the differences between the two studied types of hazardous alcohol use, drunkenness showed a nonsignificant trend for a higher association with depression and suicidality and led more often to a consultation for a mental health issue. Furthermore, frequent alcohol consumption presents slightly different associations with family-related variables comparing to drunkenness (e.g., the presence of many financial difficulties and self-employment status of the father are associated with drunkenness but not frequent alcohol consumption and—reversely—higher mother's educational level and bad relation with mother are associated with increased frequency of drinking but not drunkenness).

### 4.2. Comparison with Other Studies

Our findings are in concordance with other studies which rank Greece in the first positions among other European Countries regarding alcohol consumption but in the medium position regarding the frequency of binge drinking in adolescents [19]. Furthermore, the finding of our study that hazardous alcohol use is more frequent among older adolescents is in concordance with most relative studies [10, 11, 21] with the exception of Gatta et al. [22] who concluded that alcohol and drug use may be more common among younger adolescents. The loosening of parenting control can be considered as a possible explanation for these findings and cross-cultural differences in parental role may explain the corresponding discrepancies in international literature.

Another finding from our study was that frequent alcohol consumption and drunkenness were associated with lower school performance. This finding is in concordance with epidemiological studies from Greece and other countries, clinical studies supporting that frequent alcohol use in adolescence leads to neurocognitive impairment and studies showing that memory performance and intelligence are protective factors for developing alcohol-related problems [23–27]. In contrast to our results, Owens et al. [28] reported a positive association where more frequent alcohol consumption had been associated with higher school performance. Regarding the association between financial difficulties and alcohol-related problems our findings show that lack of financial resources may lead to increased drunkenness but not to increased frequency of alcohol consumption in adolescents. This finding is in partial concordance with a previous study from Greece that did not conclude any significant association between adolescents' regular alcohol consumption and their parents' socioeconomic status and in contrast with studies from the adult population in Greece or in other countries which support that severe financial difficulties are associated with heavy alcohol consumption [29–32]. Financial strain in the family may reduce adolescents' capacity to purchase alcohol and therefore reduce availability of alcohol but may also increase psychological distress, thus increasing the probability for binge drinking in the context of -self-medication” when alcohol is available free of charge [33, 34].

Regarding unemployment, only father's unemployment was weakly associated with increased probability for drunkenness, but with slightly decreased probability of heavy alcohol consumption, whereas mother's unemployment was associated with a lower risk for both negative alcohol-related outcomes, in contrast with a study of Torikka et al. [35]. A possible explanation for our findings is that parents' unemployment may be associated with increased time spent with their children thus increasing communication and parental control or with restricted financial resources, resulting in less availability for alcohol.

A number of studies found that higher parental educational level is associated with higher rates of adolescent alcohol use [36, 37]. This finding is confirmed in our study only for the mother's educational level. Specifically, adolescents with mothers of higher educational level were more prone to drink frequently but not more possible to get drunk, whereas father's educational level was not associated with hazardous alcohol behavior. This finding is in contrast with Arvanitidou et al. [10] who concluded that parents of adolescents who drink more frequently were less educated compared to the parents of adolescents who did not drink. Further research regarding the possible differences on norms over alcohol and parenting style in families with varying levels of parents' education or the possible “cohort-effect” on the association between adolescents alcohol consumption and parents educational level, could clarify this complex association.

Regarding the quality of relationship with the parents our findings are in concordance with studies supporting that bad communication between family members may lead to increase alcohol and drug use or towards peer-group which are prone to delinquent behavior and substance use [38–41]. Nevertheless, a recent systematic review found weak evidence for a longitudinal association between relationship with parents and alcohol use implying that the poor relationship with parents may be a result rather than a risk factor for developing alcohol use problems [42].

Finally, the association between hazardous alcohol use and depression or anxiety disorders found in our study, replicates findings from similar studies and contrast other studies concluding such an association only in early adolescence [43–46]. Although, a reverse causality explanation is possible (e.g., increased alcohol use increases the risk of a new onset of depression), evidence from large epidemiological studies support that this reverse association is less common [47]. Furthermore, the short period of exposure to alcohol during adolescence may not suffice to cause depression, as it may be the case for larger periods of exposure during adulthood. Thus, the association between hazardous alcohol consumption and mental health problems during adolescence indirectly confirms the “self-medication” hypothesis, where alcohol is used to alleviate psychological symptoms [33, 48]. Finally, the association between alcohol consumption, smoking, and cannabis use found in our study is in concordance with other studies implying a common biological and environmental causal substrate for all addictions [49].

The association of alcohol consumption and the use of health services has been poorly studied in international literature. Our finding that hazardous alcohol use is associated with increased use of general health services and mental health services (especially in drunkenness) is consistent with other studies that found that adolescents who received mental health services were more likely than those who did not, to meet the criteria for alcohol consumption disorders [50, 51]. Given the high comorbidity of alcohol use with depression and anxiety disorders this is a finding that should be studied further and has some practical clinical implications for both general doctors and mental health professionals.

## 5. Limitations

Our findings should be considered taking into account the following limitations:

- Our sample did not include students attending technical vocational school or adolescents who left the formal educational system. Therefore, our results cannot be generalized to those subpopulations.
- The cross-sectional nature of the study cannot exclude issues of reverse causality as a possible explanation for the reported associations and therefore any associations reported here should not be considered as “causal,” especially in the association between alcohol abuse and depression or poor relationship with parents.
- Our survey focused on the association between alcohol use and the common mental disorders (mainly depressive and anxiety disorders). We did not cover the full spectrum of psychiatric disorders. Psychotic disorders such as schizophrenia or severe mood disorders such as bipolar were excluded from assessment because they were expected to be rare in the school community.

## 6. Conclusion

Alcohol use in adolescence is a public health problem, strongly associated with depression and other common mental health disorders and increased use of general health services, which warrants further attention and preventive interventions. The examination of the associations that hazardous alcohol consumption in adolescence may present would help the development of more focused preventive strategies. Based on our results special attention should be paid to adolescents coming from families with bad relationship with the parents, smokers and adolescents suffering from depression or anxiety disorders or presenting more often to general health services.

Greece is a country with permissive societal norms over alcohol, which is mainly used in everyday life as a nutritional element rather than as a psychotropic intoxicant on weekends, which is more common in adolescents who originated from Northern Europe [8, 9]. Furthermore, our study was performed in a period just before the current economical austerity period in Greece. There is some evidence that economical crises may cause an increase in mental health disorders and an increase in abstinence as well as heavy alcohol use in the adulthood population [52–54]. These changes may imply modifications in the way that alcohol is used by the population and consequently in the associations of Alcohol Use Disorders (AUD) with psychosocial determinants and mental health disorders. The data on the adolescent population is scarce although crucial, as hazard rates for the onset of AUD increase rapidly during late adolescence and peak during early adolescent [55]. Therefore epidemiological assessment of alcohol use in adolescence would be very helpful in order to plan effective prevention strategies, especially in countries like Greece experiencing financial recession and, consequently, fewer available prevention resources.

## Figures and Tables

**Table 1:** Frequency of Alcohol use in 16–18 year-old adolescents in Greece by gender and type of alcohol (*N* = 2431).

	Male	Female	Total
	N^1^ (%)–(95% CI^2^)	N^1^ (%)–(95% CI^2^)	N^1^ (%)–(95% CI^2^)
*Beer or wine*			
*Never*	208 **(21.1%)** (17.7%–24.6%)	533 **(39.38%)** (35.9%–42.9%)	741 **(30.15%)** (27.6%–32.7%)
*Monthly or less*	434 **(47.5%)** (43.2–51.8)	677 **(45.42%)** (41.9%–48.9%)	1111 **(46.47%)** (43.7%–49.3%)
*At least weekly*	347 **(31,3%)** (27,4%–35.2%)	232 **(15, 2%)** (12.7% –17.7%)	579 **(23.4%)** (21.1%–25.7%)

*Spirits*			
*Never*	330 **(34,6%)** (30.5%– 38.7%)	636 **(47,1%)** (43.6% –50.6%)	966 **(40.8%)** (38%–43.5%)
*Monthly or less*	393 **(40,8%)** (36.6%–45.1%)	586 **(40%)** (36.6%–43.4%)	979 **(40.4%)** (37.7%–43.2%)
*At least weekly*	266 **(24.5%)** (21%–28%)	217 **(12.9%)** (10.7% ‐ 15.1%)	483 **(18.8%)** (16.7%–20.9%)

*Any type of alcohol*			
*Never*	148 **(14.2%)** (11.3%–17.1%)	383 **(28.7%)** ( 25.4%–32%)	531 **(21.4%)** (19.1%–23.6%)
*Monthly or less*	414 **(46.6%)** (42.3%–50.9%)	721 **(49.3%)** (45.8%–52.8%)	1135 **(47.9%)** (45.1%–50.7%)
*At least weekly*	427 **(39.2%)** (35.1%–43.3%)	338 **(21.9%)** (19.1%– 24.8%)	765 **(30.7%)** (28.1%–33.2%)

*Getting Drunk*			
*Never*	570 **(61.5%)** (57.4%–65.6%)	962 **(71%)** (68%–74%)	1532 **(66.1%)** (63.6%–68.8%)
*Once (lifetime)*	201 **(19%)** (15.7%–22.3%)	293 **(17.8%)** (15.4%–20.2%)	494 **(18.4%)** (16.4%–20.5%)
≥*2 times (lifetime)*	218 **(19.42%)** (16.20%–22.63%)	184 **(11.24%)** (9.06%–13.43%)	402 **(15.39%)** (13.43%–17.35%)

^1^Actual number of observations; percentages are weighted to take into account the stratified random sampling procedure; ^2^CI: Confidence Interval.

**Table 2:** Sociodemographic associations of frequent alcohol drinking and getting drunk in 16–18 year-old adolescents in Greece (*N* = 2431).

	Drinking alcohol at least weekly		Getting drunk ≥2 times	
	Crude OR^1^ (95% CI^3^)	Adjusted OR^2^ (95% CI^3^)	Crude OR^1^ (95% CI^3^)	Adjusted OR^2^ (95% CI^3^)
*Female gender*	**0.43 (0.34‐0.55)**	0.38^∗^**(0.29–0.48)**	**0.53 (0.39–0.71)**	0.43^∗^**(0.32–0.60)**
*Grade*				
10^th^	1.00	1.00	1.00	1.00
11^th^	1.30 (0 .93–1.83)	1.36 (0.96–1.94)	**1.66** (1.08–2.55)	**1.71** (1.11–2.65)
12^th^	**1.54** (1.09–2.17)	1.64^∗^ (1.14–2.35)	**1.71** (1.10–2.66)	1.78^∗^ (1.14–2.78)
*Parent's marital status*				
Married	1.00	1.00	1.00	1.00
Divorced/ Separated	1.57 (0.98–2.52)	1.15 (0.71–1.86)	*2.09* (1.25–3.49)	1.45 (0.87–2.41)
Widow	1.17 (0.52–2.62)	1.64 (0.62–4.32)	1.88 (0.80–4.42)	1.92 (0.72–5.16)
*Number of siblings*				
None	1.00	1.00	1.00	1.00
One	0.95 (0.62–1.44)	1.09 (0.69–.71)	0.66 (0.39–1.11)	0.85 (0.48–1.53)
Two	0.83 (0.52–1.31)	0.95 (0.57–1.57)	0.71 (0.40–1.28)	0.79 (0.41–1.54)
Three or more	0.75 (0.45–1.26)	0.91 (0.53–1.57)	0.68 (0.36–1.27)	0.71 (0.36–1.41)
*Father's educational level*				
Primary	1.00	1.00	1.00	1.00
Secondary Basic	1.49 (0.94–2.35)	1.46 (0.91–2.37)	0.80 (0.44–1.43)	1.04 (0.57–1.92)
Secondary Complete	1.24 (0.84–1.83)	1.15 (0.74–1.77)	0.86 (0.53–1.39)	1.19 (0.70–2.02)
Technological degree	1.33 (0.82–2.14)	1.11 (0.68–1.84)	0.67 (0.36–1.22)	0.82 (0.44–1.53)
University degree	1.17(0.79–1.74)	1.11 (0.69–1.78)	0.88 (0.54–1.44)	1.27 (0.73–2.20)
*Mother's educational level*				
Primary	1.00	1.00	1.00	1.00
Secondary Basic	1.47 (0.91–2.36)	1.42 (0.84–2.39)	0.80 (0.44–1.46)	0.85 (0.44–1.64)
Secondary Complete	1.37 (0.91–2.05)	1.46 (0.90–2.35)	**0.53** (0.32–0.88)	0.62 (0.34–1.12)
Technological degree	**1.96** (1.16–3.30)	**1.94** (1.08–3.50)	0.91 (0.49–1.72)	1.05 (0.50–2.20)
University degree	1.41 (0.91–2.17)	1.72 (0.99–3.00)	0.86 (0.51–1.45)	1.13 (0.56–2.25)
*Father's employment status*				
Public sector employee	1.00	1.00	1.00	1.00
Private sector employee	1.25 (0.90–1.74)	1.28 (0.88–1.86)	0.98 (0.65–1.50)	1.09 (0.71–1.69)
Self‐employed	1.21 (0.90–1.64)	1.37 (0.97–1.93)	**1.60** (1.10–2.33)	1.97^∗^ (1.29–3.00)
Retired	0.88 (0.50–1.52)	1.02 (0.57–1.82)	0.92 (0.41–2.05)	1.12 (0.48–2.59)
Unemployed/Other	0.90 (0.51–1.56)	0.64 (0.311–1.28)	1.88 (0.94–3.74)	1.21 (0.59–2.47)
*Mother's employment status*				
Public sector employee	1.00	1.00	1.00	1.00
Private sector employee	1.23 (0.86–1.78)	1.12 (0.74–1.68)	0.90 (0.58–1.41)	0.79 (0.49–1.30)
Self‐employed	0.92 (0.62–1.38)	0.91 (0.58–1.41)	0.92 (0.55–1.53)	0.80 (0.45–1.44)
Unemployed	0.79 (0.44–1.40)	0.79 (0.44–1.41)	0.80 (0.42–1.52)	0.77 (0.39–1.49)
Looks after house	0.84 (0.61–1.16)	0.90 (0.62–1.32)	0.88 (0.58–1.33)	0.93 (0.56–1.54)
Retired/Other	1.02 (0.56–1.85)	0.98 (0.53–1.83)	0.98 (0.55–1.75)	0.72 (0.34–1.50)
*Financial difficulties*				
No	1.00	1.00	1.00	1.00
Some	**1.53** (1.15–2.04)	1.52^∗^ (1.13–2.05)	1.43 (0.99–2.07)	**1.50** (1.04–2.17)
A lot	1.08 (0.75–1.57)	0.84 (0.55–1.26)	**1.59** (1.00–2.50)	1.27 (0.76–2.10)
*School performance*				
Fair	1.00	1.00	1.00	1.00
Good	**0.58** (0.36–0.96)	**0.60** (0.36–1.00)	**0.35** (0.18–0.69)	0.39^∗^ (0.19–0.80)
Very good	**0.51** (0.36–0.72)	0.51^∗^ (0.35–0.73)	**0.30** (0.20–0.46)	0.33^∗^ (0.21–0.53)
Excellent	**0.61** (0.45–0.85)	0.57^∗^ (0.41–0.79)	**0.43** (0.30–0.62)	0.45^∗^ (0.31–0.66)
*Relationship with father*				
Good/very good	1.00	1.00	1.00	1.00
Fair/bad	**2.29** (1.72–3.06)	**1.56** (1.05‐2.32)	**2.60** (1.85–3.66)	**1.97** (1.15–3.40)
*Relationship with mother*				
Good/very good	1.00	1.00	1.00	1.00
Fair/bad	**2.51** (1.83–3.45)	1.86^∗^ (1.20–2.87)	**2.24** (1.58–3.20)	1.14 (0.67–1.95)

^1^Crude OR: Unadjusted odds ratios;^ 2^Adjusted OR: Odds ratios adjusted for all other variables of the table;^ 3^CI: Confidence Interval; Bold values indicate statistical significance; Italic values indicated with an asterisk were statistically significant at the 99% level.

**Table 3:** Comorbidity of frequent alcohol drinking and getting drunk with depression, anxiety disorders and other substances use in 16–18  years-old adolescents in Greece (*N* = 2431).

Comorbid Condition	% in adolescents with drink alcohol at least weekly^1^	Odds Ratios^2^ (95% CI^3^)	% in adolescents who have been drunk ≥ 2 times^1^	Odds Ratios^2^ (95% CI^3^)
*Any psychiatric disorder*	22.8%	**1.89** (1.45–2.45)	26.35%	**2.10** (1.53–2.88)
Depressive episode	7.5%	**2.10** (1.51–2.91)	10.65%	**2.99** (2.05–4.38)
GAD^4^	13.48%	**1.73** (1.30–2.28)	15.46%	**1.87** (1.35–2.59)
Panic disorder	2.75%	1.56 (0.84–2.90)	2.29%	1.13 (0.58–2.21)
Phobias	6.48%	**1.59** (1.03–2.46)	8.36%	**2.03** (1.21–3.41)
OCD	5.22%	**1.63** (1.11–2.38)	6.99%	**2.18** (1.41–3.38)
Suicidal ideation*^5^*	4.76%	**1.82** (1.23–2.70)	6.76%	**2.65** (1.73–4.07)
Current Cigarette Smoking	27.48%	**10.67** (7.36–15.47)	41.58%	**13.49** (9.32–19.54)
Lifetime Cannabis use	10.27%	**5.22** (3.20–8.51)	20.16%	**13.94** (9.03–21.53)

*^1^ All percentages are weighted to account for the stratified random sampling; ^2^Odds ratios are adjusted for age and sex and calculated from logistic regression models with the comorbid condition as the dependent variable and the alcohol related variable as the independent variable (e.g., the odds of any psychiatric disorder was 1.89 times higher for adolescents who drink alcohol weekly compared to participants who do not drink alcohol weekly); ^3^*CI: Confidence Intervals*; ^4^GAD: Generalized Anxiety Disorder; ^5^Suicidal ideation defined as having at least thoughts that “life is not worth living” during the past week.*

**Table 4:** Use of health services use among 16–18 year-old adolescents with frequent alcohol drinking and getting drunk (*N* = 2431).

	Frequent use of health services^1^			
	For a medical reason		For psychological reasons only	
	*%^2^*	*OR^3^ (95% CI^4^)*	*%^2^*	*OR^3^ (95% CI^4^)*
*Nonhazardous use*	16%	1.00	3.10%	1.00
*Drink alcohol weekly*	21.4%	**1.44** (1.06–1.94)	4.13%	1.34 (0.70–2.57)
*Getting drunk *≥*2 times*	22.91%	**1.48** (1.03–2.13)	5.84%	**2.07** (1.09–3.93)
*Either drink weekly or getting drunk *≥*2 times*	21.27%	**1.45** (1.08–1.95)	4.54%	1.63 (0.87–3.04)

*^1^Frequent use of services defined as having visited (a) a family doctor for any medical reason ≥3 times during the past 12 months or (b) a family doctor or a mental health professional for a psychological reason at least once during the previous 12  months. ^2^All percentages are weighted to account for the stratified random sampling; ^3^Odds ratios are adjusted for age and sex and calculated from logistic regression models with frequent doctor visits as the dependent variable and hazardous alcohol use as the independent variable (e.g., the odds of frequent doctor visits for a medical reason was 1.44 times higher for participants with at least weekly alcohol consumption compared to participants with nonhazardous alcohol use); ^4^*CI: Confidence Intervals.

## Data Availability

The data used to support the findings of this study are available from the corresponding author upon request.
